# The Intestinal Microbiota Plays a Role in *Salmonella*-Induced Colitis Independent of Pathogen Colonization

**DOI:** 10.1371/journal.pone.0020338

**Published:** 2011-05-25

**Authors:** Rosana B. R. Ferreira, Navkiran Gill, Benjamin P. Willing, L. Caetano M. Antunes, Shannon L. Russell, Matthew A. Croxen, B. Brett Finlay

**Affiliations:** 1 Michael Smith Laboratories, The University of British Columbia, Vancouver, British Columbia, Canada; 2 Department of Microbiology and Immunology, The University of British Columbia, Vancouver, British Columbia, Canada; Charité-University Medicine Berlin, Germany

## Abstract

The intestinal microbiota is composed of hundreds of species of bacteria, fungi and protozoa and is critical for numerous biological processes, such as nutrient acquisition, vitamin production, and colonization resistance against bacterial pathogens. We studied the role of the intestinal microbiota on host resistance to *Salmonella enterica* serovar Typhimurium-induced colitis. Using multiple antibiotic treatments in 129S1/SvImJ mice, we showed that disruption of the intestinal microbiota alters host susceptibility to infection. Although all antibiotic treatments caused similar increases in pathogen colonization, the development of enterocolitis was seen only when streptomycin or vancomycin was used; no significant pathology was observed with the use of metronidazole. Interestingly, metronidazole-treated and infected C57BL/6 mice developed severe pathology. We hypothesized that the intestinal microbiota confers resistance to infectious colitis without affecting the ability of *S.* Typhimurium to colonize the intestine. Indeed, different antibiotic treatments caused distinct shifts in the intestinal microbiota prior to infection. Through fluorescence *in situ* hybridization, terminal restriction fragment length polymorphism, and real-time PCR, we showed that there is a strong correlation between the intestinal microbiota composition before infection and susceptibility to *Salmonella*-induced colitis. Members of the Bacteroidetes phylum were present at significantly higher levels in mice resistant to colitis. Further analysis revealed that Porphyromonadaceae levels were also increased in these mice. Conversely, there was a positive correlation between the abundance of *Lactobacillus* sp. and predisposition to colitis. Our data suggests that different members of the microbiota might be associated with *S*. Typhimurium colonization and colitis. Dissecting the mechanisms involved in resistance to infection and inflammation will be critical for the development of therapeutic and preventative measures against enteric pathogens.

## Introduction

The mammalian intestinal microbiota is a highly complex community of microorganisms that live in close association with its host. These microbes are important in numerous biological processes and are essential for health. The phyla Firmicutes and Bacteroidetes (BAC) comprise 90% of the intestinal microbiota, whereas members of the Proteobacteria, Actinobacteria, Verrucomicrobia and Cyanobacteria are present in low amounts [1]. The importance of the intestinal microbiota in human health is increasingly acknowledged and a surge in the number of research papers on the subject have been published in recent years. Imbalances of the intestinal microbiota composition have been linked to several diseases such as diabetes, inflammatory bowel disease, colorectal cancer and atopic diseases [2], [3], [4], [5].

*Salmonella enterica* serovar Typhimurium are Gram-negative, facultative intracellular pathogens that cause a wide range of human illnesses, from typhoid fever to gastroenteritis. In mice, *S*. Typhimurium normally causes a disease that resembles systemic typhoid fever. However, streptomycin treatment prior to *S.* Typhimurium infection in mice leads to a disease similar to salmonellosis in humans. Salmonellosis is marked by increased *S.* Typhimurium colonization of the intestines and a strong inflammatory response leading to colitis [6]. Recent work in our lab described that treatments with low doses of streptomycin, a broad-spectrum antibiotic, and vancomycin, an antibiotic targeting Gram-positive bacteria, altered the intestinal microbiota composition of C57BL/6 mice and increased susceptibility to *S.* Typhimurium infection and intestinal pathology, without changing overall numbers of intestinal microbiota [7].

In this study, we used several antibiotics to modify the intestinal microbiota composition of 129S1/SvImJ mice, which are more resistant to *S*. Typhimurium infection than C57BL/6 mice. We showed that there is a strong correlation between distinct subsets of the intestinal microbiota and protection against *S.* Typhimurium colonization and associated colitis in this model. While treatments with streptomycin and vancomycin increased both *S.* Typhimurium colonization and intestinal inflammation, we found that metronidazole treatment significantly increased colonization by *S.* Typhimurium without inducing colitis. However, metronidazole treatment in C57BL/6 mice increased both colonization and colitis, similar to the effects of streptomycin treatment in both mouse strains. Differences in the intestinal microbiota composition of these murine strains following metronidazole treatment and similarities between streptomycin-treated 129S1/SvImJ and metronidazole-treated C57BL/6 mice suggest that specific members of the microbiota are associated with the development of colitis. A better understanding of the role of specific members of the microbiota in resistance to infection will help facilitate the development of mechanisms to manipulate the intestinal microbiota for human benefit.

## Results

### Streptomycin, vancomycin and metronidazole treatments increase intestinal colonization by *S.* Typhimurium

**Figure 1:**
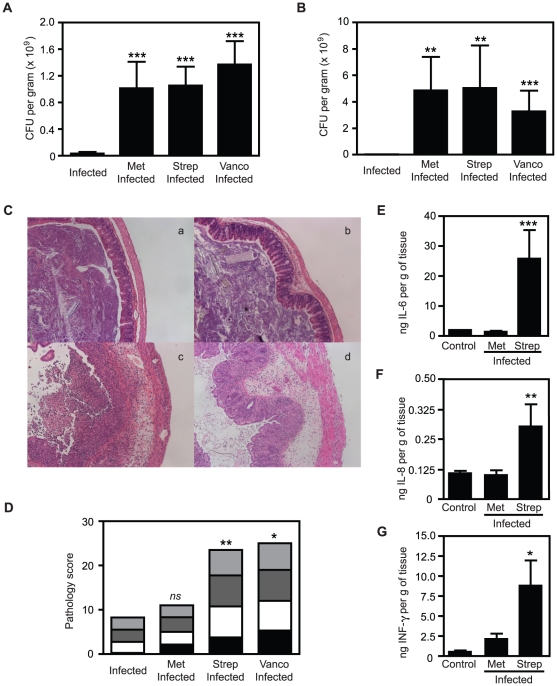
Different antibiotic treatments lead to different degrees of intestinal pathology in 129S1/SvImJ mice. A. *S*. Typhimurium levels in caeca of 129S1/SvImJ mice after 4 days of infection with or without antibiotic treatment. B. *S*. Typhimurium levels in feces of 129S1/SvImJ mice after 4 days of infection with or without antibiotic treatment. C. H&E staining of caeca of 129S1/SvImJ mice. (a). infected 129S1/SvImJ mice; (b). metronidazole-treated and infected 129S1/SvImJ mice; (c). streptomycin-treated and infected 129S1/SvImJ mice; (d). vancomycin-treated and infected 129S1/SvImJ mice. D. Pathology scores of caeca of 129S1/SvImJ mice after 4 days of infection with or without antibiotic treatment. E. Levels of IL6 in caecal samples measured by ELISA. F. Levels of IL8 in caecal samples measured by ELISA. G. Levels of IFNγ in caecal samples measured by ELISA. Met: metronidazole (750 mg/L), Strep: streptomycin (450 mg/L) and Vanco: vancomycin (50 mg/L). Black bars represent pathology scores of the intestinal lumen, white bars represent scores of the surface epithelia, dark grey bars represent scores of the mucosa and light grey bars represent scores of the submucosa of the tissue. *ns:* not significant; *: p<0.05; **: p<0.01; ***: p≤0.001. Experiments were performed three times with at least 4 mice in each group.

In order to study the impact of antibiotic treatment on host susceptibility to infection, we treated 129S1/SvImJ mice with antibiotics in their drinking water before oral infection with *S.* Typhimurium. Four days post-infection, mice were sacrificed and samples from caecum, spleen and feces were analyzed. *S*. Typhimurium caecal colonization was increased over ten-fold in streptomycin-, vancomycin- and metronidazole-treated mice compared to control-infected mice (Figure 1A). *S*. Typhimurium counts were similar between mice treated with each of the different antibiotics. Colonization levels in feces were comparable to the counts obtained in caecal samples (Figure 1B). At the systemic level (spleen), *S*. Typhimurium counts did not significantly change after antibiotic treatment compared to the infected control mice (data not shown).

### Metronidazole treatment does not increase intestinal pathology of *Salmonella*-infected 129S1/SvImJ mice

After *S*. Typhimurium infection, we observed that the intestines of infected-only and metronidazole-treated infected mice had a similar appearance, suggesting that treatment with this antibiotic did not increase inflammation following infection. However, the intestines of streptomycin- and vancomycin-treated mice appeared shorter and lacked fecal matter, classical macroscopic signs of *S*. Typhimurium-induced colitis (data not shown). In agreement with our macroscopic observations, histological evaluation of haematoxilin-eosin stained caecal tissues from metronidazole-treated and infected mice showed limited signs of inflammation (Figure 1C). On the other hand, tissues from streptomycin- and vancomycin-treated infected mice displayed markers of significant inflammation, with severe edema of the submucosa, necrotic cells and neutrophils in the lumen and widespread presence of polymorphonuclear leukocytes (PMNs) in the epithelium. The degree of tissue inflammation was further analyzed through pathological scoring of caecal samples (Figure 1D). Pathology scores were significantly different between metronidazole-treated infected mice and streptomycin and vancomycin-treated infected mice, with the latter two showing significantly stronger intestinal pathology. These results indicate that even though *S*. Typhimurium colonization levels were similar after each antibiotic treatment, the intestinal pathology observed was strikingly different. Unlike streptomycin and vancomycin, metronidazole pre-treatment does not exacerbate *S*. Typhimurium-induced colitis. In order to gain insights into the inflammation invoked by *S.* Typhimurium infection after antibiotic pre-treatment, caecal cytokine levels of 129S1/SvImJ were analyzed by ELISAs (Figure 1E, 1F and 1G). Metronidazole treatment prior to *S*. Typhimurium infection in 129S1/SvImJ mice did not significantly change IL6, IL8 or IFNγ production. However, streptomycin-treated and infected 129S1/SvImJ mice showed increased levels of the cytokines tested. These data are in agreement with the severe caecal pathology observed in these mice, as these cytokines are involved in pro-inflammatory responses.

### Metronidazole treatment increases intestinal pathology in *Salmonella*-infected C57BL/6 mice

**Figure 2:**
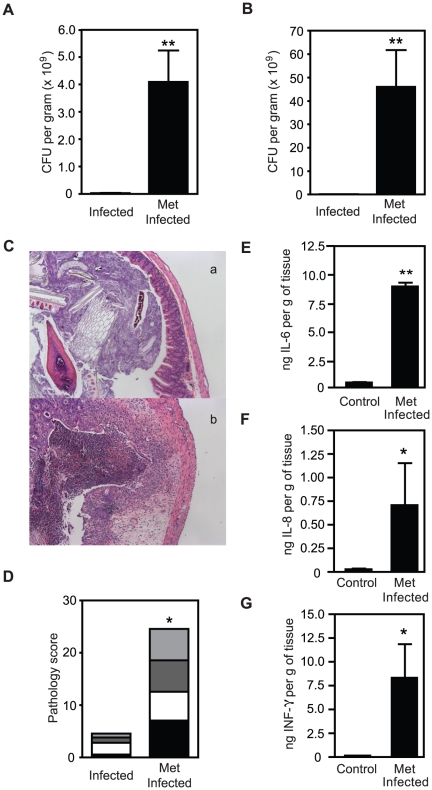
Metronidazole treatment prior to *S. Typhimurium infection induces colitis in C57BL/6 mice.* A. *S*. Typhimurium levels in caeca of C57BL/6 mice after 4 days of infection with or without antibiotic treatment. B. *S*. Typhimurium levels in feces of C57BL/6 mice after 4 days of infection with or without antibiotic treatment. C. H&E staining of caeca of C57BL/6 mice. (a). infected C57BL/6 mice; (b). metronidazole-treated and infected C57BL/6 mice. D. Pathology scores of caeca of C57BL/6 mice after 4 days of infection with or without metronidazole treatment. E. Levels of IL6 in caecal samples measured by ELISA. F. Levels of IL8 in caecal samples measured by ELISA. G. Levels of IFNγ in caecal samples measured by ELISA. Met: metronidazole (750 mg/L). Black bars represent pathology scores of the intestinal lumen, white bars represent scores of the surface epithelia, dark grey bars represent scores of the mucosa and light grey bars represent scores of the submucosa of the tissue. *: p<0.05; **: p<0.01. Experiments were performed three times with at least 4 mice in each group.

In order to further characterize the phenotype seen with metronidazole treatment, we investigated the effects of this antibiotic in C57BL/6 mice prior to infection and compared the results with those seen with 129S1/SvImJ mice. Consistent with what was observed with 129S1/SvImJ mice, metronidazole treatment of C57BL/6 animals significantly increased *S*. Typhimurium colonization of caecum and feces by over 10-fold (Figure 2A and 2B). However, contrary to our observations of metronidazole-treated 129S1/SvImJ, treatment of C57BL/6 mice with this antibiotic significantly increased intestinal pathology (Figure 2C and 2D). Cytokine levels in the ceacum of C57BL/6 mice were also analyzed (Fig 2E, 2F and 2G). Metronidazole-treated and infected mice showed increased levels of IL6, IL8 and IFNγ compared to control mice, in agreement with the pathology observed in these mice. Altogether these data indicate that the same antibiotic can have opposite effects on different strains of mice, presumably due to differences in microbiota and/or host genetic background.

### The difference in susceptibility to colitis displayed by metronidazole-treated C57BL/6 and 129S1/SvImJ mice is not due to Nramp1

One of the major differences between 129S1/SvImJ and C57BL/6 mice is their Nramp1 (Natural resistance-associated macrophage protein 1, also called Slc11a1) status, which represents a critical host defense mechanism against *S*. Typhimurium [8]. C57BL/6 mice are Nramp1 −/− and, therefore, highly susceptible to *S*. Typhimurium, succumbing to the disease within 5 days of infection. 129S1/SvImJ mice are Nramp1 +/+ and, as such, are more resistant to and generally able to control the infection. Therefore, we investigated whether the difference in susceptibility to colitis between metronidazole-treated C57BL/6 and 129S1/SvImJ mice was due to their Nramp1 status. To do so, both 129S1/SvImJ Nramp1 −/− and +/+ mice were treated with metronidazole prior to infection. Our results showed that metronidazole treatment increased *S*. Typhimurium colonization without increasing caecal inflammation in both Nramp1 −/− and +/+ mice (Figure S1). Therefore, the differences between metronidazole treated 129S1/SvImJ and C57BL/6 mice are not a result of differences in their Nramp1 status.

### The susceptibility to colitis caused by antibiotic treatment correlates with differences in the composition of the intestinal microbiota

**Figure 3:**
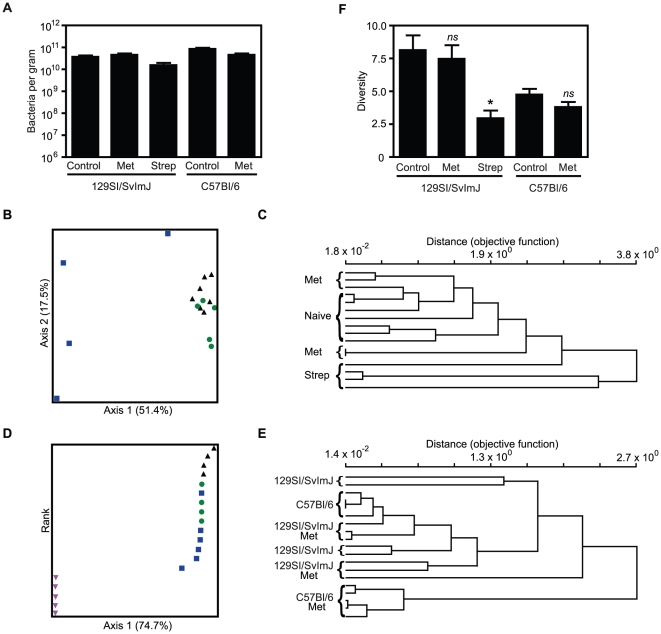
Different antibiotics have distinct effects on the composition of the intestinal microbiota. A. Total bacterial counts of fecal samples of 129S1/SvImJ and C57BL/6 uninfected mice before and after antibiotic treatments, as determined by SYBR Green staining. B. Non-metrical multidimentional scaling (NMS) ordination of Terminal restriction fragment length polymorphism (TRFLP) data of 129S1/SvImJ uninfected mice before and after streptomycin or metronidazole treatment. Black triangles: naïve 129S1/SvImJ; green circles: metronidazole-treated 129S1/SvImJ; blue squares: streptomycin-treated 129S1/SvImJ. C. Dendrogram of TRFLP data of 129S1/SvImJ mice before and after streptomycin or metronidazole treatment. D. NMS ordination of TRFLP data of 129S1/SvImJ and C57BL/6 mice before and after metronidazole treatment. Black triangles: naïve 129S1/SvImJ; green circles: naïve C57BL/6; blue squares: metronidazole-treated 129S1/SvImJ; pink inverted triangles: metronidazole-treated C57BL/6. E. Dendrogram of TRFLP data of 129S1/SvImJ and C57BL/6 mice before and after metronidazole treatment F. Microbial diversity in feces (Simpson's diversity index) before and after the antibiotic treatments. Met: metronidazole (750 mg/ L) and Strep: streptomycin (450 mg/ L). *ns*: not significant; *: p<0.05; **: p<0.01; ***: p≤0.001. Experiments were performed three times with at least 4 mice in each group.

Initially, we investigated if bacterial abundance was affected by the antibiotic treatments through SYBR green staining of fecal samples. Total intestinal bacterial numbers did not significantly change after any of the antibiotic treatments (Figure 3A), suggesting that the changes observed are not simply due to a decrease in total commensal numbers. We also analyzed the total number of viable bacteria in fecal samples under aerobic and anaerobic conditions. No significant changes were observed when the total number of colonies grown under both conditions was combined. However, a significant change was observed in the total number of colonies grown under aerobiosis in streptomycin-treated 129S1/SvImJ mice (Figure S2). Terminal-restriction fragment length polymorphism (TRFLP) was used to assess the bacterial 16S rRNA composition of the feces of antibiotic-treated uninfected mice. The use of fecal samples allowed us to collect multiple samples from the same mouse at different points of the treatment without having to euthanize the animal. However, to ensure that the results obtained with these samples were representative of what occurred in the caecum, caecal microbiota was also analyzed by TRFLP. No significant differences between the fecal and caecal samples of mice from the same treatment were observed (data not shown). The composition of each fecal sample was plotted using non-metric multidimensional scaling (NMS) ordination based on Sorensen distance measure to assess overall changes in microbial community composition between treatment groups [9]. Both metronidazole and streptomycin treatment in 129S1/SvImJ mice resulted in changes in microbial composition (p<0.05, p<0.005, respectively) (Figure 3B and 3C). However, streptomycin and metronidazole had distinct effects on the fecal microbial composition (p*<*0.001) , causing changes in the microbiota that were positively and negatively correlated with caecal pathology upon infection, respectively. The effect of metronidazole treatment on the fecal microbiota of C57BL/6 mice was also compared to metronidazole-treated 129S1/SvlmJ mice (Figure 3D and 3E). Despite the fact that the fecal microbiota of the two mouse strains was relatively similar before antibiotic treatment, metronidazole had a more marked effect on the microbiota of C57BL/6 mice (p<0.005). Again, these changes correlate well with the pathology observed after *S*. Typhimurium infection, as streptomycin-treated 129S1/SvImJ and metronidazole-treated C57BL/6 mice exhibited stronger colitis compared to metronidazole-treated 129S1/SvImJ mice. The microbial diversity, as assessed by Simpson's index of diversity [10], indicate that streptomycin treatment decreased diversity (p<0.05) in 129S1/SvImJ mice, whereas metronidazole did not (Figure 3F). Interestingly, metronidazole treatment of C57BL/6 mice did not significantly alter the microbial diversity. Although this may seem contradictory to our hypothesis that these parameters of microbiota structure are responsible for susceptibility to colitis, it is important to note that the diversity of C57BL/6 mice was much lower than that observed in 129S1/SvImJ mice. It is possible that this is an important determinant that may be predisposing C57BL/6 mice to antibiotic-induced colitis.

### Specific subsets of the intestinal microbiota can be correlated with protection against colitis

**Figure 4:**
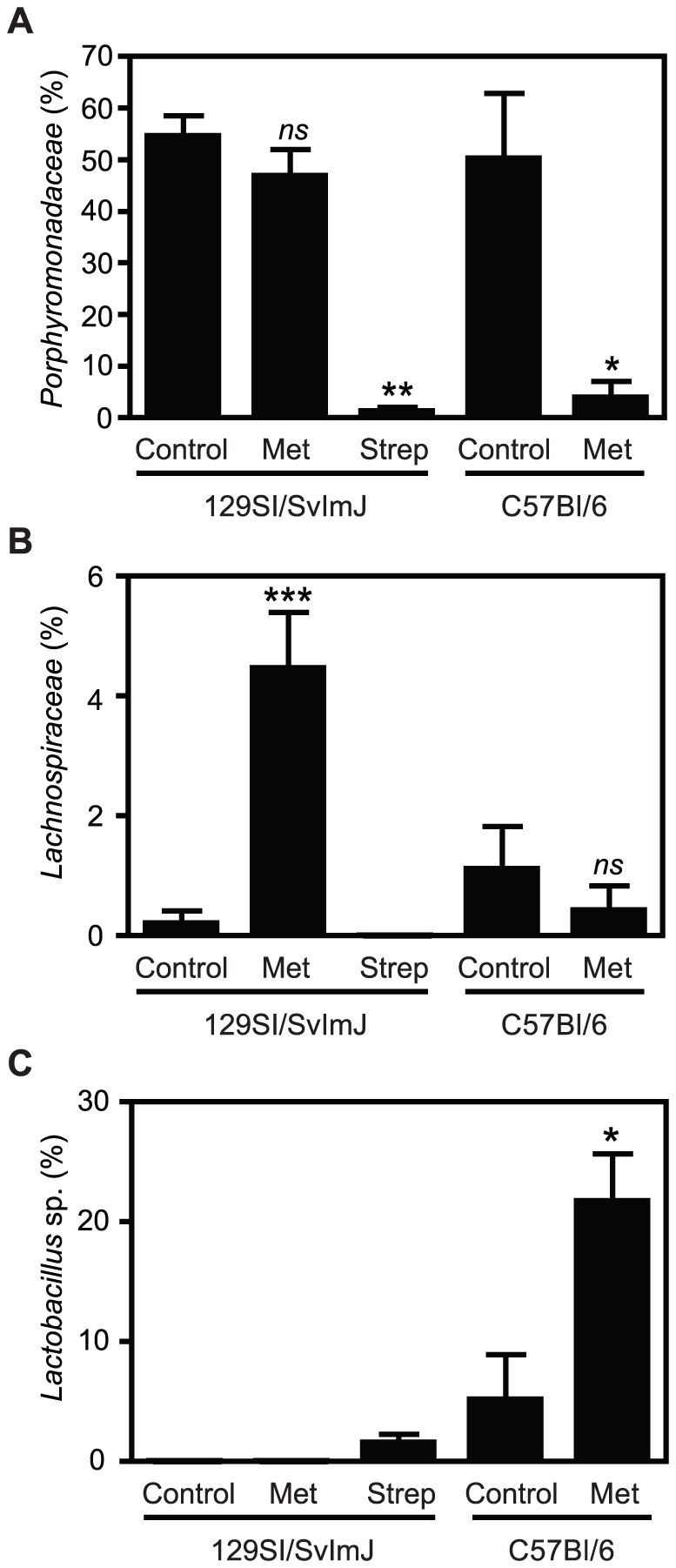
Specific members of the microbiota are involved in predisposition to colitis. The contribution of individual peaks to the total peak intensity of each sample was determined through TRFLP analyses. A. Porphyromonadaceae (TFR 83–87). B. Lachnospiraceae (TRF 154). C. *Lactobacillus* sp. (TRF 187–188). *ns*: not significant; *: p<0.05; **: p<0.01; ***: p≤0.001. Experiments were performed three times with at least 4 mice in each group.

By comparing the abundance of individual terminal restriction fragment (TRF) lengths in protective versus non-protective conditions it was possible to identify a small subset of TRFs that were negatively and positively correlated with susceptibility to colitis. Clone libraries of 16S rRNA genes were generated and screened with restriction digestion to allow for phylogenetic assignment of TRFs of interest. TRFs in the range of 83 to 87 bp remained abundant after metronidazole treatment of 129S1/SvImJ mice, but were significantly decreased in colitis-susceptible mice, thus representing potentially protective organisms (Figure 4A). Clones corresponding to these TRFs were all classified within the family Porphyromonadaceae from the Bacteroidetes phylum. Conversely, TRF188, identified as *Lactobacillus*, was present at increased levels in streptomycin-treated 129S1/SvImJ and metronidazole-treated C57BL/6, but was not detectable after metronidazole treatment of 129S1/SvImJ, and was thus positively correlated with colitis susceptibility (Figure 4C). We also found that TRF154 was present at significantly higher levels in metronizadole-treated 129S1/SvImJ, but was unaffected by other treatments (Figure 4B). Clones corresponding to this TRF were classified within the family Lachnospiraceae. It is important to note that an attempt to grow viable bacteria from the fecal microbiota of the different groups in selective media showed no correlation with the phenotypes observed in mice (data not shown). This highlights the importance of DNA-based methodologies to thoroughly analyze the composition of the intestinal microbiota.

**Figure 5:**
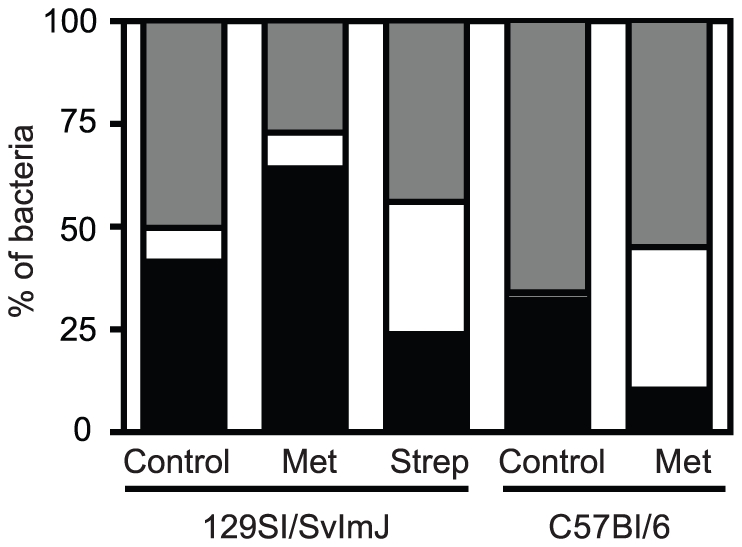
Predisposition to colitis can be associated with phylum-level changes in the composition of the intestinal microbiota caused by antibiotic treatments. Fluorescent *in situ* hybridization (FISH) of fecal samples of 129S1/SvImJ and C57BL/6 mice before and after antibiotic treatment. Met: metronidazole (750 mg/ L) and Strep: streptomycin (450 mg/ L). Black bars represent the percentage of bacteria belonging to the BAC phyla. White bars represent the percentage of bacteria belonging to the GAM phyla and gray bars represent the percentage of Firmicutes and other groups. Experiments were performed three times with at least 4 mice in each group.

Fluorescent *in situ* hybridization (FISH) was also performed to further analyze the microbial composition of the intestinal microbiota at the phylum level. For this purpose, we used specific probes to calculate the percentages of Bacteroidetes (BAC), Gammaproteobacteria (GAM) and Firmicutes and others (FIM) in the intestinal microbiota. Consistent with the TRFLP data, metronidazole treatment caused an increase in the BAC numbers in 129S1/SvImJ mice (Figure 5) whereas a depletion of this microbial group was seen in streptomycin-treated 129S1/SvImJ and metronidazole-treated C57BL/6 mice. Therefore, BAC abundance could correlate with resistance to colitis. On the other hand, increased GAM numbers were observed in colitis-susceptible mice prior to infection and seemed to correlate with increased susceptibility to colitis.

**Figure 6:**
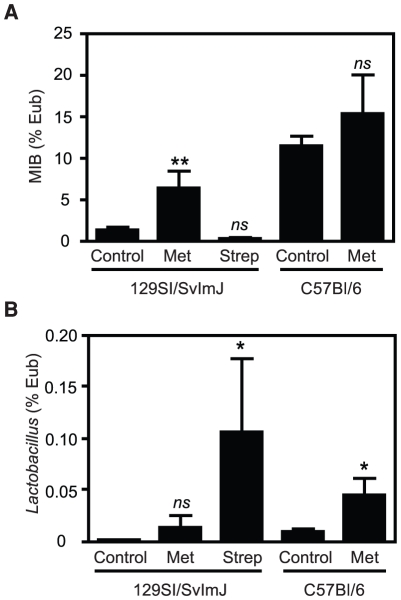
MIB and *Lactobacillus* sp. levels are associated to predisposition to *S*. Typhimurium-induced colitis. A. Levels of MIB on fecal samples of 129S1/SvImJ and C57BL/6 mice before and after antibiotic treatment, as determined by RT-PCR. B. Levels of *Lactobacillus* on fecal samples of 129S1/SvImJ and C57BL/6 mice before and after antibiotic treatment, as determined by RT-PCR. *ns*: not significant; *: p<0.05; **: p<0.01; ***: p≤0.001. Experiments were performed three times with at least 4 mice in each group.

Real-time PCR was used to confirm the results of TRFLP and clone-library data, and to further characterize the abundance of specific bacterial subsets that were below detection levels of community profiling techniques. Because of the differences in the Bacteroidetes numbers between the groups predisposed or resistant to colitis, we investigated the levels of Mouse Intestinal Bacteria (MIB) [11], a subset of Porphyromonadaceae, in these mice. We also confirmed the results obtained by TRFLP, which showed a prevalence of TRF188, identified as *Lactobacillus* sp., in samples of mice predisposed to colitis. We observed that MIB levels did not significantly change after streptomycin treatment of 129S1/SvImJ or metronidazole treatment of C57BL/6 mice, both models that are predisposed to colitis. In contrast, MIB levels were significantly increased after metronidazole treatment of 129S1/SvImJ (Figure 6A). The opposite was seen when we analyzed *Lactobacillus* numbers, which increased after streptomycin (colitis-inducing) but not metronidazole (colitis non-inducing) treatment of 129S1/SvImJ (Figure 6B).

Because of the recent data on metronidazole treatment increasing *Bifidobacteria* presence in the colon of rats [12], the presence of *Bifidobacteria* was also determined in our models. *Bifidobacteria* were below detection levels in 129S1/SvImJ mice, but significantly increased after metronidazole treatment of C57BL/6 (data not shown). Additionally, segmented filamentous bacteria (SFB) have also been shown to be an important component of the intestinal microbiota, increasing resistance to the mouse pathogen *Citrobacter rodentium* [13], [14]. In our models, the presence of SFB could not be detected in any of the samples analyzed (data not shown), suggesting that these bacteria are not involved in the development of colitis described here.

## Discussion

Changes in the intestinal microbiota have been described as important players in a vast number of both intestinal and extra-intestinal diseases [15], [16]. It has been shown that the composition of the intestinal microbiota protects mice from *S*. Typhimurium colonization in the gut [7]. In the present study, we describe that the inflammatory process elicited during *S*. Typhimurium-induced colitis is not due only to high levels of *S*. Typhimurium encountered in the intestine after antibiotic treatment. Although 129S1/SvImJ mice treated with metronidazole prior to infection had high levels of *S*. Typhimurium intestinal colonization, they did not develop severe caecal pathology and did not show increased levels of pro-inflammatory cytokines. On the other hand, mice treated with streptomycin had high levels of *S*. Typhimurium as well as marked signs of infectious colitis. We also showed that metronidazole induces distinct caecal pathologies and inflammatory responses in 129S1/SvImJ and C57BL/6 mice, which coincided with differences in microbiota populations. This indicates that differences in host microbiota might explain the differences in the outcome of infection observed in our study. The results obtained herein reflect the effect of the specific antibiotic treatments described. Different antibiotic doses and different treatment regimens may have had different outcomes in the intestinal pathologies and host responses observed. Metronidazole is a nitroimidazole antibiotic effective against anaerobes that is routinely used in the treatment of inflammatory bowel diseases (IBD) and has been shown to decrease postoperative recurrence in Crohn's disease (CD) patients [17], diseases linked to uncontrolled immune responses to the intestinal microbiota. However, the effectiveness of the use of metronidazole in the treatment of CD is still under debate [18]. Some studies have shown a positive effect of this drug on CD treatment [17], [19], [20], whereas others show no significant difference between metronidazole treatment and the placebo [21], [22].

The two strains of mice used in our models, 129S1/SvImJ and C57BL/6, are commonly used as resistant and susceptible typhoid infection models, respectively [23], [24]. One difference in these mice is that C57BL/6 mice possess a point mutation in the gene encoding Nramp1, leading to a non-functional protein. As a result, these mice are susceptible to *S*. Typhimurium, and succumb to uncontrolled bacterial replication [23]. 129S1/SvImJ, on the other hand, express Nramp1 and are able to survive *S*. Typhimurium infection [23]. Therefore, we investigated whether Nramp1 status was the determining factor in the differences in susceptibility to colitis upon metronidazole treatment displayed by these two mouse strains. The intestinal pathology of infected 129S1/SvImJ and an isogenic mutant of Nramp1 −/− after metronidazole treatment was analyzed and no differences were observed, indicating that Nramp1 is not involved in this phenomenon. Although one of the major differences between 129S1/SvImJ and C57BL/6 mouse strains is Nramp1 expression, there are other genetic differences with known impacts on inflammatory responses, such as C5 deficiency and Vnn haplotype differences that may be playing a role in these phenotypic differences [25], [26], [27], [28]. We hypothesize that differences in the intestinal microbiota between these two models may be one of the important factors in determining susceptibility to colitis.

In this work, we explored the intestinal microbiota composition as a player in the development of *S*. Typhimurium-induced colitis in mice. We demonstrated that significant differences in the composition of the intestinal microbiota during antibiotic treatment of the two different mouse strains are strongly associated with predisposition to colitis. By comparing streptomycin versus metronidazole treatment of 129S1/SvImJ and metronidazole treatment of C57BL/6 versus 129S1/SvImJ, we were able to circumvent issues related to host genetics and potential direct effects of antibiotics on the host, thus substantiating the role of the microbiota in infection. Our data suggests that specific members of the microbiota are linked to protection against inflammation but do not affect *S*. Typhimurium colonization. These members are overrepresented when 129S1/SvImJ mice are treated with metronidazole, but they decrease or are unchanged after streptomycin treatment in these mice or metronidazole treatment in C57BL/6 mice. Our data indicates that they belong to the Bacteroidetes (BAC) phylum, particularly to the Porphyromonadaceae family, which is represented here by Mouse Intestinal Bacteria (MIB). MIB are part of a recently discovered operational taxonomic unit (OTU), which is very closely related to *Bacteroides*. It is present in all parts of the intestinal tract of mice [11] and is also found in lower numbers in human feces [29]. In addition to the increased numbers of MIB in mice resistant to *S*. Typhimurium-induced colitis, we also found bacterial groups that seem correlated with susceptibility to colitis. These include *Lactobacillus* sp., which belong to the Firmicutes phylum and are present in the intestinal tract of many animals [11]. Several studies have shown a positive effect of different species of *Lactobacillus* on protecting against intestinal pathophysiology [30]. However, most clinical trials showed that these bacteria were not able to induce remission in IBD patients [31], [32], [33]. Our data indicates that *Lactobacillus* sp. levels are significantly increased in streptomycin-treated 129S1/SvImJ and metronidazole-treated C57BL/6 mice, both predisposed to infectious colitis. In agreement with this finding, a recent study revealed that certain prebiotics, which stimulate the growth of *Lactobacillus* sp., among other bacteria, increase the severity of *S*. Typhimurium infection in mice [34]. The increase of *Lactobacillus* sp. occurred only in instance of reduced Bacteroidetes, and therefore it is not possible to determine which one of these events is responsible for the control of the inflammatory response to *Salmonella* infection observed herein. Furthermore, the fact that *Lactobacillus* sp. levels in control C57BL/6 mice (resistant to colitis) are higher than in streptomycin-treated 129S1/SvImJ mice (susceptible to colitis) (Figure 4C) indicates an indirect effect of these species on susceptibility to colitis. Although a strong correlation is shown here, further studies are necessary to prove a definitive causal link between these bacteria and *S*. Typhimurium-induced colitis.

Recently, it was shown that metronidazole alters the gut microbiota of rats and reduces basal oxidative stress to proteins in colonic tissue. It has also been demonstrated that this antibiotic increases the thickness of the mucus layer and the presence of *Bifidobacteria*, which would decrease intestinal inflammation in IBD patients [12]. In our model, metronidazole treatment induces protection against *S*. Typhimurium-induced intestinal inflammation only in one of the mouse strains tested. It is important to note that treatment with this antibiotic did not increase the presence of *Bifidobacteria* in these mice, suggesting that the potentially protective role of *Bifidobacteria* in rats is distinct from the phenotype observed in our study.

Our data strongly support the notion that specific microbial components of the intestinal microbiota determine predisposition to colitis. Since it has been known for several decades that the intestinal microbiota protects against *S.* Typhimurium colonization, it has been generally assumed that the protection against colitis was a consequence of reduced levels of *S.* Typhimurium in the intestines of untreated mice. However, our study adds another layer of complexity to the role of the microbiota in protection against infectious agents. We now know that the protection against colonization and inflammation are two independent events, and that different subsets of the microbiota are responsible for each aspect of disease resistance. It has been know that certain commensals produce molecules that can affect the host immune system and protect against intestinal inflammation [35], [36] and this might be the case in our study. Dissecting the potentially numerous unidentified roles of microbial members of the intestinal biota will be fundamental in our quest towards understanding the complexities of the human body and its relationship with our microbe partners.

## Materials and Methods

### Ethics Statement

All animal experiments were performed in strict accordance with the guidelines of the University of British Columbia Animal Care Committee and the Canadian Council on the Use of Laboratory Animals. The protocol was approved by the UBC Animal Care Committee (Certificate number: A09-0168). The mice were euthanized by CO_2_ asphyxiation and all efforts were made to minimize suffering.

### Mice

Female, age-matched (6–8 weeks old) C57BL/6 and 129Sv/ImJ Nramp1 −/− and Nramp1 +/+ [8], [37] mice were obtained from Jackson Laboratory (Bar Harbor, ME) or from a breeding colony maintained at the Wesbrook Animal Unit at The University of British Columbia. Nramp1 −/− mice have a null allele at the Nramp1 locus (the Nramp1 gene was interrupted by an inserted neomycin cassette), and thus, mRNA transcripts are absent. Mice were fed a standard sterile chow diet (Laboratory Rodent Diet 5001, Purina Mills, St. Louis, Missouri) *ad libitum* throughout the experiments.

### Bacterial Strains

*Salmonella enterica* serovar Typhimurium strain SL1344 [38] was grown overnight at 37°C with shaking (200 rpm) in Luria-Bertani (LB) broth. This strain is resistant to streptomycin, metronidazole and vancomycin.

### Antibiotic treatments and *S*. Typhimurium infection

Mice were treated with 450 mg/L of streptomycin (Sigma) or 50 mg/L vancomycin (Sigma) in drinking water for 2 days or 750 mg/L of metronidazole (Sigma) in drinking water for 7 days. Antibiotics were dissolved in filter-sterilized water; control mice were given water without antibiotics. After the antibiotic treatment period, regular drinking water was returned and mice were infected with ∼2.7×10^8^ *S*. Typhimurium in LB broth by oral gavage. Uninfected control mice were given 100 µL of sterile LB broth. At 4 days post-infection, mice were euthanized by CO_2_ asphyxiation and samples were harvested aseptically for further analysis. Antibiotic-treated, uninfected mice were euthanized by CO_2_ asphyxiation after the treatment period.

### Sample collection and assessment of *S*. Typhimurium colonization

Caeca, spleen and feces were collected in 1 mL of sterile phosphate-buffered saline (PBS). The samples were kept on ice and homogenized with an MM 301 Mixer Mill (Retsch, Newton, PA). Serial dilutions of the homogenates were plated on LB agar plates supplemented with 100 µg/mL of streptomycin to enumerate *S*. Typhimurium. Plates were incubated overnight at 37°C and colonies were counted after incubation. Fecal samples were also collected throughout the experiments and kept at −80°C for further microbiota analysis.

### Histopathology

Caecal samples were collected and fixed in 10% neutral buffered formalin overnight and then placed into 75% ethanol. Fixed tissues were embedded in paraffin and cut into 5 µm sections. Tissues were stained with hematoxylin and eosin (H&E) using standard techniques by Wax-it Histology Services (Vancouver, BC, Canada) and UBC Histology Laboratory. Pathological scores were assigned as previously described [39]. Briefly, the scoring system was performed as follows: for lumen, sum of empty (score 0), necrotic cells (scant 1, moderate 2, dense 3) and PMNs (scant 2, moderate 2, severe 3); for surface epithelium, sum of no pathological change (score 0), regenerative changes (mild 1, moderate 2, severe 3), desquamation (patchy 1, diffuse 2), PMNs (score 1), ulceration (score 1); for mucosa, sum of no pathological change (score 0), crypt abscesses (rare 1, moderate 2, abundant 3), presence of mucinous plugs (score 1) and granulation tissue (score 1); for submucosa, sum of no pathological changes (score 0), mononuclear cell infiltrate (one small aggregate 0, more than one 1, large aggregates plus increase single cells 1), PMN infiltrate (single 1, aggregates 2) and edema (moderate 1, severe 2). Pathology Images were taken using a Zeiss Axioskop 2 microscope.

### Microbiota analysis

Total bacterial counts from fecal samples were performed using SYBR Green staining as described previously [40]. Briefly, a 1∶10 dilution of the homogenized fecal samples were fixed and stored in 3.7% formalin at 4°C. Two µL were stained with 0.25 µL of SYBR Green (Invitrogen) and viewed with an Olympus 1x81 microscope. Cells were counted from three randomly chosen fields and the numbers averaged. Counts were corrected based on volume used, dilution and the known diameter of the microscope field. Fluorescent *in situ* hybridization (FISH) was performed as previously described [7]. Five to 100 µL of sample was hybridized to 250 ng of the general EUB338 probe (5′-GCT GCC TCC CGT AGG AGT-3′), fluorescently labeled with Texas Red [41] and 250 ng of either the CFB286 (5′-TCC TCT CAG AAC CCC TAC-3′) or GAM42a (5′-GCC TTC CCA CAT CGT TT-3′) probes [42] labeled with fluorescein, then viewed and counted as described for SYBR staining. The percentage compositions of Bacteroidetes and Gammaproteobacteria were calculated by dividing the numbers obtained for each phylum by the total number of Eubacteria. The remaining bacteria were defined as Firmicutes and others, as previously described [7].

### Enumeration of viable bacteria from the fecal microbiota

Fecal samples were collected in 1 mL of PBS with 0.5% cysteine, homogenized with an MM 301 Mixer Mill and immediately taken to an anaerobic chamber. Serial dilutions were performed in PBS containing 0.5% cysteine and plated in Tryptose blood agar base, Reinforced clostridial agar, Rogosa and Anaerobe basal agar (Oxoid). Tryptose blood agar base and Anaerobe basal agar were supplemented with 7% sheep blood (Cedarlane). Plates were incubated under aerobic (Tryptose blood agar base) or anaerobic conditions (all the media) for 48h at 37°C and colonies were counted after incubation.

### Terminal-Restriction Fragment Length Polymorphism (TRFLP)

Total DNA was isolated from fecal and caecal samples using the QIAamp DNA stool kit (Qiagen) according to the manufacturer's instructions with the addition of a bead-beating step. Polymerase Chain Reaction (PCR) was performed to amplify bacterial 16S rDNA present in fecal samples using broad-range bacterial primers Bact8F(FAM) (5′-AGA GTT TGA TCM TGG CTC AG-3′) [43] labeled with 6-carboxyfluorescein on the 5′-end and 926R (5′-CCG TCA ATT CCT TTR AGT TT-3′) [44]. The PCR products were digested with MspI (New England Biolabs, Ipswich, MA) and sent to analysis at NAPS (Michael Smith Laboratories, Vancouver, BC). Electropherograms were processed using Gene Marker v.1.75 software (State College, PA) and relative peak areas of terminal restriction fragments corresponding to sizes between 50–600 bp were analyzed. Data were plotted using PC-ORD5 software (MjM Software, Gleneden Beach, OR).

### Cloning and Sequencing

Clone libraries were generated for the phylogenetic classification of TRFs of interest. PCR amplified 16S rRNA genes from DNA isolated from feces were cloned into the TA pCR® 4 TOPO vector and inserted into *Escherichia coli* chemically competent cells (Invitrogen). Transformants were screened by TRFLP as previously described, and those coinciding with peaks of interest were sequenced. Sequences were classified using the naïve Bayesian rRNA classifier in RDP [45].

### Quantitative Real-Time PCR (RT-PCR)

The abundance of specific intestinal bacterial groups in DNA extracted from fecal samples was measured by RT-PCR on a 7500 Fast Real-Time System (Applied Biosystems, Foster City, CA). The reactions were performed using Quantitec SYBR Green mastermix (Qiagen) with the following group-specific 16S rDNA primers (IDT, Coralville, IA). Eubacteria (all bacteria): UniF340 ACT CCT ACG GGA GGC AGC AGT and UniR514 ATT ACC GCG GCT GCT GGC (60°C) [46]; Mouse Intestinal Bacteria (MIB): UNI516F CCA GCA GCC GCG GTA ATA and ambMIBR677 CGG CAT TCC GCR TAC TTC TC (60°C) ([46] and this study); *Lactobacillus*/*Lactococcus*: LabF362 ACG AGT AGG GAA ATC TTC CA and LabR677 CAC CGC TAC ACA TGG AG (56°C) [46]; Segmented Filamentous Bacteria (SFB): SFB736F GAC GCT GAG GCA TGA GAG CAT and SFB844R GAC GGC ACG GAT TGT TAT TCA (58°C) [46]; *Bifidobacterium* BIF164 GGG TGG TAA TGC CGG ATG and BIF662 CCA CCG TTA CAC CGG GAA (62°C) [47]. Eubacteria primers were used to amplify a segment of the 16S rDNA to determine the total amount of bacteria in each sample. The RT-PCR program started with two steps of 50°C for 2 minutes and 95°C for 10 minutes, followed by 40 cycles of 95°C for 30 seconds and 60°C for 1 minute, when data was acquired. The same DNA sample was amplified using the group-specific primers described above observing their different annealing temperatures at the final step. Group-specific bacterial numbers were determined as a percentage of the numbers obtained with the Eubacteria primers.

### ELISAs

Caecum homogenates were centrifuged twice at 16,000 g, and the supernatants were stored at −80°C. The levels of interleukin-8 (IL8), interleukin-6 (IL6), interferon gamma (IFNγ), tumor necrosis factor alpha (TNFα) (R&D System, Minneapolis, MN) and interleukin-22 (IL22) (eBioscience, San Diego, CA) were measured by enzyme–linked immunosorbent assays (ELISAs) according to the manufacturers' instructions.

### Statistical Analysis

Statistical significance was calculated using the Mann-Whitney test with a 95% confidence interval. All analyses were performed using GraphPad Prism version 4.0 (GraphPad Software, San Diego, CA). The results were expressed as mean values with standard errors of the means. Microbial community composition was plotted using non-metric multidimensional scaling (NMS) ordination based on Sorensen distance measure, and significance between treatments was tested using the multiple response permutation procedure (MRPP) in PC-ORD5 software (Gleneden Beach, OR).

## Supporting Information

Figure S1 **Nramp1 is not responsible for the differences in intestinal pathology between 129S1/SvImJ and C57BL/6 mice observed following metronidazole treatment and infection.** A. *S*. Typhimurium levels in the caeca of Nramp1 +/+ and Nramp1 −/− 129S1/SvImJ mice after 4 days of infection with or without antibiotic treatment. B. *S*. Typhimurium levels in feces of Nramp1 +/+ and Nramp1 −/− 129S1/SvImJ mice after 4 days of infection with or without antibiotic treatment. C. H&E staining of caeca of Nramp1 +/+ and Nramp1 −/− 129S1/SvImJ mice. (a). infected Nramp1 +/+ 129S1/SvImJ mice; (b). metronidazole-treated and infected Nramp1 +/+ 129S1/SvImJ mice; (c). infected Nramp1 −/− 129S1/SvImJ mice; (d). metronidazole-treated and infected Nramp1 −/− 129S1/SvImJ mice. D. Pathology scores of caeca of Nramp1 +/+ and Nramp1 −/− 129S1/SvImJ mice after 4 days of infection with or without antibiotic treatment. Met: metronidazole (750 mg/L). Black bars represent pathology scores of the intestinal lumen, white bars represent scores of the surface epithelia, dark grey bars represent scores of the mucosa and light grey bars represent scores of the submucosa of the tissue. *ns*: not significant; *: p<0.05; **: p<0.01. Experiments were performed three times with at least 4 mice in each group.
(EPS)

Figure S2 **Determination of viable bacteria in fecal samples incubated in aerobiosis and anaerobiosis in Tryptose blood agar base.** Total bacterial counts in control, metronidazole-treated or streptomycin-treated uninfected 129S1/SvImJ mice and control and metronidazole-treated uninfected C57BL/6 mice. Aerobiosis (black bars) and Anaerobiosis (white bars).
(EPS)

## References

[1] Eckburg PB, Bik EM, Bernstein CN, Purdom E, Dethlefsen L (2005). Diversity of the human intestinal microbial
flora.. Science.

[2] Frank DN, St Amand AL, Feldman RA, Boedeker EC, Harpaz N (2007). Molecular-phylogenetic characterization of microbial community
imbalances in human inflammatory bowel diseases.. Proc Natl Acad Sci U S A.

[3] Turnbaugh PJ, Ley RE, Mahowald MA, Magrini V, Mardis ER (2006). An obesity-associated gut microbiome with increased capacity for
energy harvest.. Nature.

[4] Penders J, Thijs C, van den Brandt PA, Kummeling I, Snijders B (2007). Gut microbiota composition and development of atopic
manifestations in infancy: the KOALA Birth Cohort Study.. Gut.

[5] Yang L, Pei Z (2006). Bacteria, inflammation, and colon cancer.. World J Gastroenterol.

[6] Barthel M, Hapfelmeier S, Quintanilla-Martinez L, Kremer M, Rohde M (2003). Pretreatment of mice with streptomycin provides a Salmonella
enterica serovar Typhimurium colitis model that allows analysis of both
pathogen and host.. Infect Immun.

[7] Sekirov I, Tam NM, Jogova M, Robertson ML, Li Y (2008). Antibiotic-induced perturbations of the intestinal microbiota
alter host susceptibility to enteric infection.. Infect Immun.

[8] Valdez Y, Grassl GA, Guttman JA, Coburn B, Gros P (2009). Nramp1 drives an accelerated inflammatory response during
Salmonella-induced colitis in mice.. Cell Microbiol.

[9] Culman SW, Gauch HG, Blackwood CB, Thies JE (2008). Analysis of T-RFLP data using analysis of variance and ordination
methods: a comparative study.. J Microbiol Methods.

[10] Begon M, Harper J. L, Townsend C.R (1996). Ecology: Individuals, Populations, and
Communities..

[11] Salzman NH, de Jong H, Paterson Y, Harmsen HJ, Welling GW (2002). Analysis of 16S libraries of mouse gastrointestinal microflora
reveals a large new group of mouse intestinal bacteria.. Microbiology.

[12] Pelissier MA, Vasquez N, Balamurugan R, Pereira E, Dossou-Yovo F (2010). Metronidazole effects on microbiota and mucus layer thickness in
the rat gut.. FEMS Microbiol Ecol.

[13] Ivanov, II, Atarashi K, Manel N, Brodie EL, Shima T (2009). Induction of intestinal Th17 cells by segmented filamentous
bacteria.. Cell.

[14] Gaboriau-Routhiau V, Rakotobe S, Lecuyer E, Mulder I, Lan A (2009). The key role of segmented filamentous bacteria in the coordinated
maturation of gut helper T cell responses.. Immunity.

[15] Sekirov I, Russell SL, Antunes LC, Finlay BB (2010). Gut microbiota in health and disease.. Physiol Rev.

[16] Fujimura KE, Slusher NA, Cabana MD, Lynch SV (2010). Role of the gut microbiota in defining human
health.. Expert Rev Anti Infect Ther.

[17] Rutgeerts P, Hiele M, Geboes K, Peeters M, Penninckx F (1995). Controlled trial of metronidazole treatment for prevention of
Crohn's recurrence after ileal resection.. Gastroenterology.

[18] Gionchetti P, Rizzello F, Lammers KM, Morselli C, Sollazzi L (2006). Antibiotics and probiotics in treatment of inflammatory bowel
disease.. World J Gastroenterol.

[19] Ursing B, Alm T, Barany F, Bergelin I, Ganrot-Norlin K (1982). A comparative study of metronidazole and sulfasalazine for active
Crohn's disease: the cooperative Crohn's disease study in Sweden.
II. Result.. Gastroenterology.

[20] Sutherland L, Singleton J, Sessions J, Hanauer S, Krawitt E (1991). Double blind, placebo controlled trial of metronidazole in
Crohn's disease.. Gut.

[21] Blichfeldt P, Blomhoff JP, Myhre E, Gjone E (1978). Metronidazole in Crohn's disease. A double blind cross-over
clinical trial.. Scand J Gastroenterol.

[22] Ambrose NS, Allan RN, Keighley MR, Burdon DW, Youngs D (1985). Antibiotic therapy for treatment in relapse of intestinal
Crohn's disease. A prospective randomized study.. Dis Colon Rectum.

[23] Roy MF, Malo D (2002). Genetic regulation of host responses to Salmonella infection in
mice.. Genes Immun.

[24] Valdez Y, Diehl GE, Vallance BA, Grassl GA, Guttman JA (2008). Nramp1 expression by dendritic cells modulates inflammatory
responses during Salmonella Typhimurium infection.. Cell Microbiol.

[25] Mullick A, Elias M, Picard S, Bourget L, Jovcevski O (2004). Dysregulated inflammatory response to Candida albicans in a
C5-deficient mouse strain.. Infect Immun.

[26] Tuite A, Elias M, Picard S, Mullick A, Gros P (2005). Genetic control of susceptibility to Candida albicans in
susceptible A/J and resistant C57BL/6J mice.. Genes Immun.

[27] Min-Oo G, Fortin A, Pitari G, Tam M, Stevenson MM (2007). Complex genetic control of susceptibility to malaria: positional
cloning of the Char9 locus.. J Exp Med.

[28] Patel SN, Berghout J, Lovegrove FE, Ayi K, Conroy A (2008). C5 deficiency and C5a or C5aR blockade protects against cerebral
malaria.. J Exp Med.

[29] Kibe R, Sakamoto M, Yokota H, Benno Y (2007). Characterization of the inhabitancy of mouse intestinal bacteria
(MIB) in rodents and humans by real-time PCR with group-specific
primers.. Microbiol Immunol.

[30] Damaskos D, Kolios G (2008). Probiotics and prebiotics in inflammatory bowel disease:
microflora 'on the scope'.. Br J Clin Pharmacol.

[31] Zocco MA, dal Verme LZ, Cremonini F, Piscaglia AC, Nista EC (2006). Efficacy of Lactobacillus GG in maintaining remission of
ulcerative colitis.. Aliment Pharmacol Ther.

[32] Prantera C, Scribano ML, Falasco G, Andreoli A, Luzi C (2002). Ineffectiveness of probiotics in preventing recurrence after
curative resection for Crohn's disease: a randomised controlled trial
with Lactobacillus GG.. Gut.

[33] Marteau P, Lemann M, Seksik P, Laharie D, Colombel JF (2006). Ineffectiveness of Lactobacillus johnsonii LA1 for prophylaxis of
postoperative recurrence in Crohn's disease: a randomised, double
blind, placebo controlled GETAID trial.. Gut.

[34] Petersen A, Heegaard PM, Pedersen AL, Andersen JB, Sorensen RB (2009). Some putative prebiotics increase the severity of Salmonella
enterica serovar Typhimurium infection in mice.. BMC Microbiol.

[35] Mazmanian SK, Round JL, Kasper DL (2008). A microbial symbiosis factor prevents intestinal inflammatory
disease.. Nature.

[36] Round JL, Mazmanian SK (2010). Inducible Foxp3+ regulatory T-cell development by a
commensal bacterium of the intestinal microbiota.. Proc Natl Acad Sci U S A.

[37] Vidal S, Gros P, Skamene E (1995). Natural resistance to infection with intracellular parasites:
molecular genetics identifies Nramp1 as the Bcg/Ity/Lsh
locus.. J Leukoc Biol.

[38] Hoiseth SK, Stocker BA (1981). Aromatic-dependent Salmonella typhimurium are non-virulent and
effective as live vaccines.. Nature.

[39] Coburn B, Li Y, Owen D, Vallance BA, Finlay BB (2005). Salmonella enterica serovar Typhimurium pathogenicity island 2 is
necessary for complete virulence in a mouse model of infectious
enterocolitis.. Infect Immun.

[40] Lupp C, Robertson ML, Wickham ME, Sekirov I, Champion OL (2007). Host-mediated inflammation disrupts the intestinal microbiota and
promotes the overgrowth of Enterobacteriaceae.. Cell Host Microbe.

[41] Amann RI, Krumholz L, Stahl DA (1990). Fluorescent-oligonucleotide probing of whole cells for
determinative, phylogenetic, and environmental studies in
microbiology.. J Bacteriol.

[42] Manz W AR, Ludwig W, Wagner W, Schleifer KH (1992). Phylogenetic oligodeoxynucleotide probes for the major subclasses
of Proteobacteria: Problems and solutions.. Syst Appl Microbiol.

[43] Edwards U, Rogall T, Blocker H, Emde M, Bottger EC (1989). Isolation and direct complete nucleotide determination of entire
genes. Characterization of a gene coding for 16S ribosomal
RNA.. Nucleic Acids Res.

[44] Muyzer G, de Waal EC, Uitterlinden AG (1993). Profiling of complex microbial populations by denaturing gradient
gel electrophoresis analysis of polymerase chain reaction-amplified genes
coding for 16S rRNA.. Appl Environ Microbiol.

[45] Wang Q, Garrity GM, Tiedje JM, Cole JR (2007). Naive Bayesian classifier for rapid assignment of rRNA sequences
into the new bacterial taxonomy.. Appl Environ Microbiol.

[46] Barman M, Unold D, Shifley K, Amir E, Hung K (2008). Enteric salmonellosis disrupts the microbial ecology of the
murine gastrointestinal tract.. Infect Immun.

[47] Langendijk PS, Schut F, Jansen GJ, Raangs GC, Kamphuis GR (1995). Quantitative fluorescence in situ hybridization of
Bifidobacterium spp. with genus-specific 16S rRNA-targeted probes and its
application in fecal samples.. Appl Environ Microbiol.

